# Different regions of synaptic vesicle membrane regulate VAMP2 conformation for the SNARE assembly

**DOI:** 10.1038/s41467-020-15270-4

**Published:** 2020-03-24

**Authors:** Chuchu Wang, Jia Tu, Shengnan Zhang, Bin Cai, Zhenying Liu, Shouqiao Hou, Qinglu Zhong, Xiao Hu, Wenbin Liu, Guohui Li, Zhijun Liu, Lin He, Jiajie Diao, Zheng-Jiang Zhu, Dan Li, Cong Liu

**Affiliations:** 1 Interdisciplinary Research Center on Biology and Chemistry, Shanghai Institute of Organic Chemistry, Chinese Academy of Sciences, Shanghai, 201210 China; 2 University of Chinese Academy of Sciences, Beijing, 100049 China; 3 Department of Cancer Biology, University of Cincinnati College of Medicine, Cincinnati, OH 45267 USA; 4 Laboratory of Molecular Modeling and Design, State Key Laboratory of Molecular Reaction Dynamics, Dalian Institute of Chemical Physics, Chinese Academy of Sciences, 457 Zhongshan Road, Dalian, 116023 China; 5 National Facility for Protein Science in Shanghai, ZhangJiang Lab, Shanghai Advanced Research Institute, Chinese Academy of Sciences, Shanghai, 201210 China; 6 Bio-X Institutes, Key Laboratory for the Genetics of Developmental and Neuropsychiatric Disorders, Ministry of Education, Shanghai Jiao Tong University, Shanghai, 200030 China; 7 Bio-X-Renji Hospital Research Center, Renji Hospital, School of Medicine, Shanghai Jiao Tong University, Shanghai, 200240 China

**Keywords:** Lipidomics, Single-molecule biophysics, Solution-state NMR

## Abstract

Vesicle associated membrane protein 2 (VAMP2/synaptobrevin2), a core SNARE protein residing on synaptic vesicles (SVs), forms helix bundles with syntaxin-1 and SNAP25 for the SNARE assembly. Prior to the SNARE assembly, the structure of VAMP2 is unclear. Here, by using in-cell NMR spectroscopy, we describe the dynamic membrane association of VAMP2 SNARE motif in mammalian cells, and the structural change of VAMP2 upon the change of intracellular lipid environment. We analyze the lipid compositions of the SV membrane by mass-spectrometry-based lipidomic profiling, and further reveal that VAMP2 forms distinctive conformations in different membrane regions. In contrast to the non-raft region, the membrane region of cholesterol-rich lipid raft markedly weakens the membrane association of VAMP2 SNARE motif, which releases the SNARE motif and facilitates the SNARE assembly. Our work reveals the regulation of different membrane regions on VAMP2 structure and sheds light on the spatial regulation of SNARE assembly.

## Abstract (web summary)

Vesicle associated membrane protein 2 (VAMP2) contains a conserved SNARE motif that interacts with syntaxin-1 and SNAP25 for SNARE complex assembly. Here authors use in-cell NMR spectroscopy to describe the dynamic membrane association of VAMP2 SNARE motif in mammalian cells at the atomic resolution.

## Introduction

**Fig. 1:**
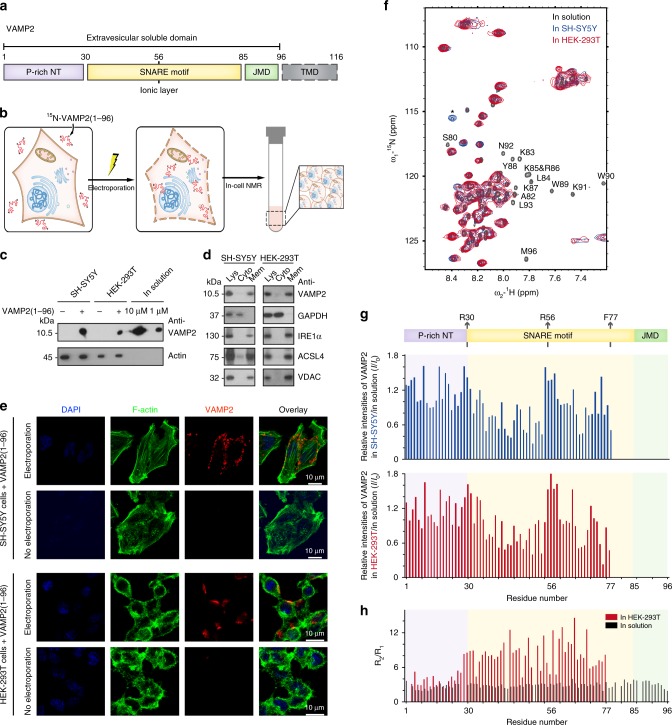
Membrane association of VAMP2 extravesicular domain in mammalian cells by in-cell NMR spectroscopy. **a** Domain organization of VAMP2. P-rich NT: proline-rich N-terminal domain. JMD: juxta-membrane domain. TMD: transmembrane domain. R56 is conserved in the VAMP family which forms the zero ionic layer in the SNARE complex. **b** Scheme of in-cell NMR sample preparation. NMR-visible ^15^N-VAMP2(1–96) was delivered into mammalian cells by electroporation. Un-delivered proteins were washed off before NMR signal acquiring. **c** Estimation of VAMP2 quantity in cells by immunoblotting. Cells prepared for NMR were diluted 10 times before loading on gels. Known concentrations of VAMP2(1–96) proteins were loaded as standards. **d** Sub-cellular localization of VAMP2(1–96) by fractionation and immunoblotting. Fractions of the total lysate (Lys), cytosol (Cyto) and membrane (Mem) were validated by immunoblotting with antibodies of GAPDH, IRE1α, ACSL4, and VDAC to indicate cytosol, endoplasmic reticulum membrane, plasma membrane-associated membrane, and mitochondrial membrane, respectively. **e** Sub-cellular localization of VAMP2(1–96) by immunofluorescence staining. F-actin filaments beneath cell membranes were stained by FITC-phalloidin. Nuclei were stained by DAPI. **f** Overlay of 2D ^1^H-^15^N NMR spectra of VAMP2(1–96) in solution (black), SH-SY5Y cells (blue) and HEK-293T cells (red). Disappeared crosspeaks in cells were denoted. *The peak was not able to be assigned. **g** Residue-resolved relative NMR intensity ratios (*I*/*I*_0_) of VAMP2 (1–96) in cells to that in solution. Domain organization of VAMP2 extravesicular domain is indicated. **h** Residue-resolved ratios of ^15^N transverse (R_2_, s^−1^) to longitudinal (R_1_, s^−1^) relaxation rates of VAMP2(1–96) in HEK-293T cells (red) and in solution (black). Source data are provided as a Source Data file.

Soluble N-ethylmaleimide-sensitive-factor attachment receptor (SNARE) complex is a macromolecular machinery, which is largely involved in membrane fusion processes, in particular the fusion of synaptic vesicle (SV) membrane with pre-synaptic plasma membrane to release neurotransmitters^1^. The assembly of SNARE complex in neurons is driven by the formation of a stable four-helix bundle between VAMP2 (also known as synaptobrevin2), syntaxin-1, and SNAP25, and modulated by multiple factors including proteins (e.g., synaptotagmin-1^2^, Munc18^3^, complexin^4^), lipids (e.g., sphingosine^5^, PIP2^6^) and metabolic ions (e.g., calcium^7^). Among the core SNARE proteins, VAMP2 resides on the SV membrane, while the other two are on the plasma membrane. VAMP2 is composed of an extravesicular soluble domain and a C-terminal transmembrane domain (Fig. 1a). The soluble domain is intrinsically disordered and composed of an N-terminal proline-rich domain, a SNARE motif and a juxta-membrane domain. It is reported that the soluble domain of VAMP2 is unstructured in solution^8^ and on the lipid nanodisc^9^. While, in other lipid environments, the SNARE motif and juxta-membrane domain can associate with lipids, and this association varies largely from transient interactions to induce the formation of α-helical structures as proceeding from lipid bilayers to bicelle and micelle environments^10,11^. However, little is known about the structure and membrane association of VAMP2 soluble domain before vesicle docking in the native environment.

In-cell NMR spectroscopy is a cutting-edge technology to obtain the structural and dynamic information of proteins inside living cells. Recently, it has been applied to the structural study of proteins, e.g. α-synuclein^12^ and superoxide dismutase 1^13^, in the cytosol of human cells. To capture the NMR signals, high levels of isotope-labeled proteins are required. To meet this requirement, one approach is to directly express isotope-labeled proteins in cells^14^. An alternative approach is to deliver the exogenous isotope-labeled proteins into cells^15–17^. Recently, Theillet et al. developed an effective approach to deliver isotope-labeled α-synuclein into human cells by electroporation^12^, which is potentially a general technique for the delivery of intrinsically disordered proteins. In this work, we deliver the intrinsically disordered N-terminal cytosolic tail of VAMP2 into human cells by electroporation and observe that it associates with membranes. In particular, the SNARE motif exhibits a dynamic interaction with native membranes, which can be adjusted by the change of cellular cholesterol levels. We further dissect that the membrane association and activity of VAMP2 SNARE motif are elegantly tuned by the different regions of SV membrane, which adds a spatial dimension on the complex modulation of SNARE machinery.

## Results

### In-cell membrane binding of VAMP2 extravesicular domain

To study the structure of VAMP2 in the native environment, we conducted in-cell NMR spectroscopy (Fig. 1b). We prepared ^15^N-labeled VAMP2(1–96) and electroporated it into HEK-293T cells and neuronal SH-SY5Y cells to cellular concentrations of ~10 μM and ~80 μM, respectively (Fig. 1c), which are comparable to the physiological concentration of VAMP2 (10–100 μM) within synaptosomes of neurons^18^. The half-life of delivered VAMP2 in cells was about 18 h (Supplementary Fig. 1). Interestingly, immunoblot showed that delivered VAMP2 is dominantly populated in the membrane fraction of the total cell lysates (Fig. 1d). Confocal microscopy further visualized that the delivered VAMP2 distributed along with the cell membrane skeleton in both cell types (Fig. 1e), which is in sharp comparison to α-synuclein, that was identified to be evenly distributed in the cytosol in the previous in-cell NMR study^12^ as well as in our experiment (Supplementary Fig. 2). These results demonstrate that, lacking of the transmembrane domain, VAMP2 remains associated with membranes in cells.

Next, we used SOFAST-HMQC pulse sequence to collect the signals of ^15^N-VAMP2(1–96) in cells. VAMP2 in HEK-293T cells exhibited a similar spectrum in terms of chemical shift and intensity, to that in SH-SY5Y cells (Fig. 1f, g), indicating that VAMP2 adopts a conserved conformation in these two different cell types. The residue-specific NMR crosspeaks of VAMP2 in cells, in comparison with those in solution, exhibited non-uniformly signal broadening without obvious chemical shift perturbations, indicating that different regions of VAMP2 may interact divergently with their cellular partners. The signal changes of VAMP2 in cells were not come from the crowding intracellular environment since crowding agents caused general signal attenuations of VAMP2, rather than regional signal changes (Supplementary Fig. 3). The 2D ^1^H-^15^N NMR spectra showed that in the intracellular environment, the signals of residues 78–96, which covers the juxta-membrane domain and a short C-terminal region of VAMP2 SNARE motif, completely disappeared (Fig. 1f, g). Deletion of the region VAMP2(78–96) resulted in a weakened membrane association with ~25% of total VAMP2 released into the cytosol (Supplementary Fig. 4), suggesting that the NMR signal missing of residues 78–96 in cells may result from membrane binding. NMR signals of the major region of the SNARE motif also exhibited significant signal attenuations (Fig. 1f, g). ^15^N transverse to longitudinal relaxation rates (R_2_/R_1_) further showed that residues 35–78 of the SNARE motif is less flexible and more ordered in cells than in solution (Fig. 1h and Supplementary Fig. 5). Truncation of the C-terminal half of the SNARE motif together with the juxta-membrane domain (VAMP2(60–96)), nearly completely abolished the membrane association of VAMP2 (Supplementary Fig. 4), which indicates that besides the juxta-membrane domain, the SNARE motif is also critical for the membrane association of VAMP2. Note that the conserved residue R56, which forms a zero ionic layer in the assembly of *trans*- to *cis*- SNARE complex^19,20^, exhibited no large signal changes (Fig. 1f, g). The N-terminal proline-rich domain also showed no significant NMR signal change in cells and in solution (Fig. 1f, g).

In addition, considering the membrane-binding property of VAMP2, to rule out the possibility that VAMP2 just stuck on the outer surface of cells, rather than entering inside, we conducted the same process of sample preparation yet without electroporation. The 2D ^1^H-^15^N NMR spectrum showed few signals (Supplementary Fig. 6), which confirms that after carefully washing during sample preparation, little VAMP2 remained outside cells, either on cell surface or in solution. We also confirmed that during NMR signal acquirement, no detectable VAMP2(1–96) or VAMP2 (1–59) leaked out from cells by immunoblotting (Supplementary Fig. 7). Thus, these results confirm that the in-cell NMR signals are derived from the membrane-associated ^15^N-VAMP2 in cells.

Taken together, these data show that different regions of VAMP2 extravesicular domain exhibit distinctive membrane-binding properties: C-terminal residues 78–96 that compose the juxta-membrane domain and a short region of SNARE motif, have a strong interaction with cell membranes; the majority of SNARE motif dynamically interacts with membranes; while, the N-terminal proline-rich domain poorly interacts with membranes.

### Cholesterol level regulates VAMP2 conformation in cells

**Fig. 2:**
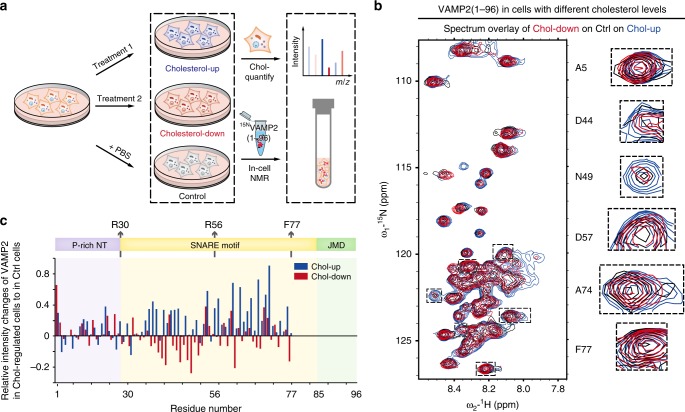
Residue-specific structural change of VAMP2(1–96) in cholesterol up- and down-regulated cells. **a** Scheme of experimental design for studying the conformations of VAMP2 in cholesterol-level-regulated cells. Treatments 1 and 2 for increasing and decreasing cellular cholesterol levels were noted in the Methods section. **b** Overlay of 2D ^1^H-^15^N NMR spectra of VAMP2(1–96) in cholesterol up-regulated (Chol-up, blue), down-regulated (Chol-down, red) and control (Ctrl, black) HEK-293T cells at the same contour levels. Representative crosspeaks were enlarged on the right. **c** Relative NMR signal intensity changes of VAMP2(1–96) in Chol-up (blue) and Chol-down (red) cells to those in control cells. Details of data processing are described in the Methods section. Source data are provided as a Source Data file.

Given the dynamic interaction of VAMP2 SNARE motif with cell membranes, we asked whether the intracellular membrane environment may regulate the membrane association of VAMP2. Previous studies suggested that SANRE proteins are recruited on cholesterol-rich lipid-raft microdomains for SNARE complex assembly^21,22^, thus we manipulated the cholesterol level of the mammalian cells following an established approach^23^ (Fig. 2a). We were able to up- and down-regulate the cellular cholesterol level in HEK-293T cells by ~60%, respectively, measured by the absolute quantification using liquid chromatography with mass spectrometry (LC-MS) and a fluorometric method (Supplementary Fig. 8). We next transported ^15^N-VAMP2(1–96) into cholesterol upregulated and downregulated cells by electroporation, respectively (Fig. 2a). Comparable amounts of VAMP2 were delivered into the cholesterol-regulated cells and the untreated cells (Supplementary Fig. 9a). We also confirmed that the cholesterol manipulation did not change the membrane localization of VAMP2 in cells (Supplementary Fig. 9b). The 2D ^1^H-^15^N NMR spectra showed that the intensities of VAMP2 signals, but not the chemical shifts, changed in correlation with the intracellular cholesterol levels (Fig. 2b). Notably, as the cholesterol level increased, the intensities of VAMP2 SNARE motif increased as well (Fig. 2c), suggesting a weakened association of the SNARE motif with cell membranes. Conversely, as the cholesterol level decreased, the intensities of SNARE motif decreased (Fig. 2c), suggesting an enhanced membrane association. In contrast, the flexible N-terminal domain of VAMP2 showed arbitrary intensity changes in cholesterol manipulated cells (Fig. 2c).

### Different regions of SV membrane regulate VAMP2 function

**Fig. 3:**
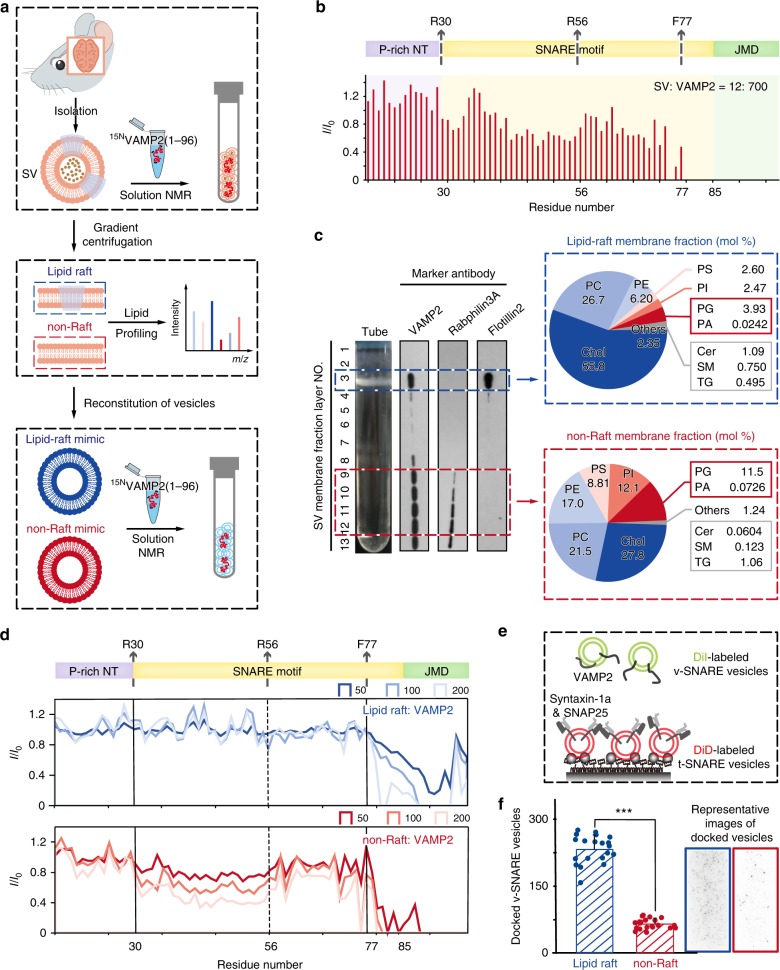
Conformational transition of VAMP2 on membrane subdomains of synaptic vesicles. **a** Scheme of experimental design for the structural study of VAMP2 on SV membrane. Upper: SVs isolated from mouse brain were used for NMR titration of VAMP2(1–96) at the physiological ratio. Middle: natural SV membranes were divided into lipid-raft and non-raft membranes by sucrose gradient sedimentation which were analyzed by quantitative lipidomic profiling. Lower: lipid-raft- and non-raft-mimicking vesicles were reconstituted with natural-sourced lipids to titrate VAMP2(1–96). **b** Residue-resolved NMR signal intensity ratios (*I*/*I*_0_) of VAMP2(1–96) titrated by SVs to that in solution. The molar ratio of SV to VAMP2(1–96) is indicated. **c** Left: distribution of endogenous VAMP2 on SV membrane. SV membranes were fractionated into 13 layers which were collected as lipid raft (layer 3) and non-raft (layers 9–12) according to flotillin2 (lipid raft membrane protein) and rabphilin3A (non-raft membrane protein). Right: lipid compositions of lipid-raft and non-raft membranes by MS-based lipidomic profiling. Chol: cholesterol; PC: phosphatidylcholine; PE: phosphatidylethanolamine; PS: phosphatidylserine; PI: phosphatidylinositol; PG: phosphatidylglycerol; PA: phosphatidic acid; Cer: ceramide; SM: sphingomyelin; TG: triacylglycerol. **d** Residue-resolved NMR signal intensity ratios (*I*/*I*_0_) of VAMP2(1–96) titrated by lipid-raft-mimicking (blue) or non-raft-mimicking (red) vesicles to that in solution at indicated lipid/protein molar ratios. **e** Scheme of single-vesicle docking assay. A saturated layer of DiD-labeled (red) t-SNARE vesicles carrying syntaxin-1a and SNAP25 was immobilized on the imaging surface. Free DiI-labeled (green) v-SNARE vesicles, reconstituted with full-length VAMP2, were injected into the system. Green laser illumination imaged the v-vesicles that docked on t-vesicles through SNARE complex formation. **f** Images on the right are representative fluorescence images of the single-vesicle docking assay. The bar graph on the left shows the numbers of lipid-raft- and non-raft-mimicking v-vesicles that docked on t-vesicles. Error bars are standard deviations from 20 random imaging locations in the same sample channel. *** indicates *p*-value < 0.001 by One-way analysis of variance (ANOVA) with Tukey Test. Source data are provided as a Source Data file.

Given that cholesterols could alter the lipid distribution of cell membranes^24^, the results derived from in-cell NMR experiments evoked us to speculate that the conformation of VAMP2 may vary in different regions of the SV membrane. Thus, we isolated SVs from mouse brains to titrate ^15^N-VAMP2(1–96) (Fig. 3a, upper). The molar ratio of VAMP2 to SV was 700:12 which is close to that within the synaptic bouton^25^. The 2D NMR spectrum of VAMP2 exhibited no obvious chemical shift deviations in the presence of SVs comparing with those in SH-SY5Y and HEK-293T cells (Supplementary Fig. 10). The *I*/*I*_0_ analysis showed a similar lipid binding pattern of VAMP2 in the presence of SVs (Fig. 3b) and in the cellular environment (Fig. 1g), with an increasing binding affinity from the N-terminal to the C-terminal. Specifically, regions Q38-L54 and D64-F77 of the SNARE motif exhibited significant intensity attenuations, suggesting a transient lipid binding of the SNARE motif of VAMP2.

Next, we fractionated SV membranes into cholesterol-enriched lipid-raft and non-raft fractions by sucrose gradient centrifugation. (Fig. 3a, middle, and Fig. 3c, left). Immunoblot showed that the endogenous VAMP2 presented in both the lipid-raft and non-raft fractions (Fig. 3c, left). To investigate the conformation of VAMP2 in these two different membrane regions, we first quantitatively characterized the lipid compositions of the lipid-raft and non-raft fractions by using MS-based lipidomic profiling (Fig. 3a, middle, Fig. 3c, right and Supplementary Data 1). The result showed that both lipid-raft and non-raft membranes are dominantly composed of glycerol-phospholipids (including PC, PE, PS, PI, PG, and PA) and cholesterol. Based on the lipidomic analyses of SV membrane subdomains, we used natural-source cholesterol and glycerol-phospholipids to reconstruct vesicles mimicking the lipid compositions of the lipid-raft and non-raft membranes, respectively (Supplementary Fig. 11). Then, we titrated them to ^15^N-VAMP2(1–96) and found a remarkable difference as VAMP2 binds to these two different membranes (Fig. 3a, lower, Fig. 3d and Supplementary Fig. 12). Overall, VAMP2 bound much tighter with the non-raft vesicle than with the lipid-raft vesicle. As VAMP2 binds to the non-raft vesicle, the SNARE motif, especially the N-terminal half of the motif, exhibited enhanced interaction with membranes as the concentration of non-raft vesicle increased (Fig. 3d, lower). In contrast, as VAMP2 binds to the lipid-raft vesicle, the SNARE motif of VAMP2 barely interacted with membranes (Fig. 3d, upper).

We next asked whether the conformational difference of VAMP2 on membrane lipid raft and non-raft influences the SNARE assembly. Thus, we reconstituted full-length VAMP2 on lipid-raft or non-raft vesicles as v-SNARE vesicles, and reconstituted syntaxin-1a and SNAP25 on lipid-raft or non-raft vesicles as t-SNARE vesicles (Fig. 3e). Stable assembly of these three proteins into the SNARE complex leads to the docking of a v-vesicle to the immobilized t-vesicle, which could be monitored by the single-vesicle fluorescence microscopy. The result showed that the v-vesicle mimicking lipid raft presented a significantly higher docking efficiency than that mimicking the non-raft (Fig. 3f).

Altogether, these results demonstrate that VAMP2, especially the SNARE motif, exhibits distinctive conformations in different regions of the SV membrane. As localizing on the cholesterol-rich lipid rafts, the SNARE motif is free of membrane binding and readily to associate with its homologous motifs of other core SNARE proteins. While, as localizing in the non-raft region of SV membrane, the SNARE motif is more associated with membrane and less active for SNARE assembly.

### Acidic lipids strengthen the membrane binding of VAMP2

**Fig. 4:**
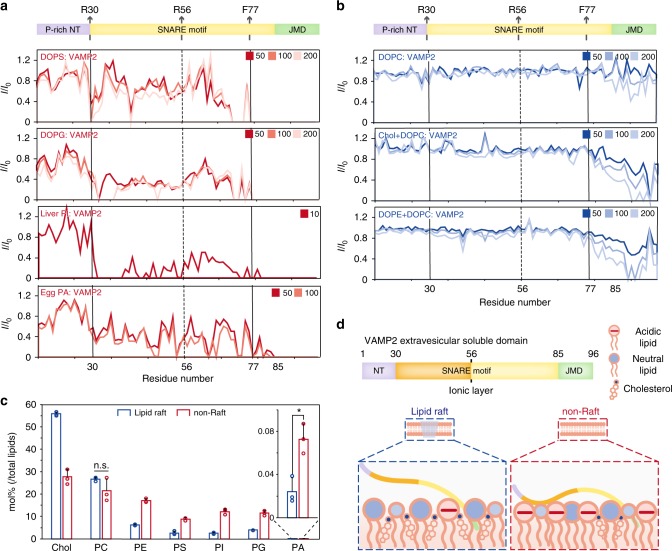
VAMP2 SNARE motif sensors the electrostatic property of membrane surface. **a** Residue-resolved NMR signal intensity ratios (*I*/*I*_0_) of VAMP2(1–96) titrated by acidic liposomes (DOPS, DOPG, liver PI, and egg PA) to that in solution at indicated lipid/protein molar ratios. **b** Residue-resolved NMR signal intensity ratios (*I*/*I*_0_) of VAMP2(1–96) titrated by neutral liposomes (DOPC, cholesterol/DOPC (1/1, mol/mol) and DOPE/DOPC (1/1, mol/mol)) to that in solution at indicated lipid/protein molar ratios. **c** Comparisons of individual lipid classes in lipid-raft and non-raft SV. Error bars are standard deviations from three biological replicates. The data were analyzed by Student’s *t*-test. The *p*-values of all lipid subclasses, except for PC and PA, are less than 0.001. n.s. represents not significant. * indicates *p*-value < 0.05. **d** Schematic presentation of VAMP2 extravesicular domain on lipid-raft and non-raft membranes. Source data are provided as a Source Data file.

To investigate the mechanism underlying the different conformations of VAMP2 in different membrane regions, we tested the individual lipid subclasses of SV membrane for their influences on the structure of VAMP2. The result of lipid analysis showed that the SV membrane mainly consists of cholesterol and glycol-phospholipids including neutral lipids (PC and PE) and acidic lipids (PS, PI, PG, and PA) (Fig. 3c, right). Thus, we titrated VAMP2(1–96) with each individual lipid and monitored their interactions with VAMP2. The NMR data showed that VAMP2 exhibited strong interactions with the negatively-charged phospholipids (Fig. 4a). A regional binding was also observed for the binding of VAMP2 to negatively-charged lipids, which is similar to that observed in cells and in the binding of VAMP2 to SVs (Figs. 4a, 1g, and 3b). In contrast, except for the juxta-membrane domain, VAMP2 showed no significant interaction with cholesterol or the neutral phospholipids (Fig. 4b). In addition, lipid profiling showed that the cholesterol-rich lipid rafts contain significantly less negatively-charged lipids than the non-raft membranes (Fig. 4c). Thus, these results indicate that the conformation of VAMP2 could be defined by the different electrostatic property of the different regions of SV membrane (Fig. 4d).

## Discussion

**Fig. 5:**
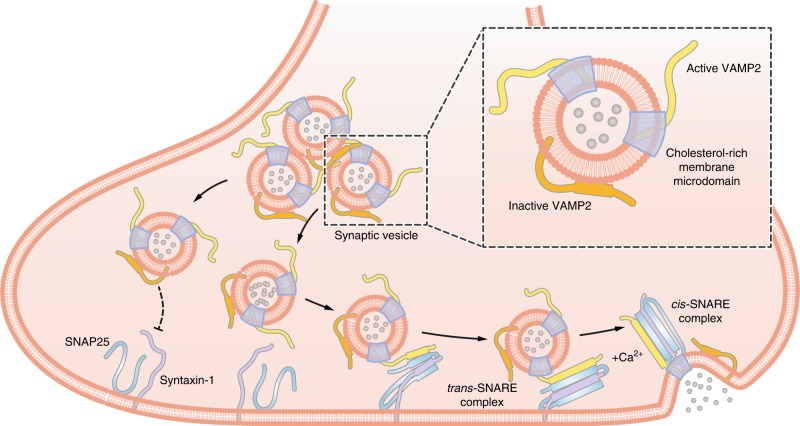
Hypothetic models of VAMP2 conformations and engagement in SNARE complex assembly for neurotransmitter release. The extravesicular domain of VAMP2 adopts distinctive conformations in different membrane regions. On the cholesterol-rich microdomains, it is less associated with SV membrane and thus more active to engage in calcium-evoked SNARE assembly and neurotransmitter release. In contrast, in other regions of SV membrane, it tends to hibernate on the membrane with an increased content of α-helical conformation which is relatively inactive in SNARE assembly.

In-cell NMR spectroscopy is a cutting-edge technology that provides residue-specific structural information of proteins in live cells^26^. In this work, we performed the in-cell NMR to study a membrane-associated protein—VAMP2, and captured its structural changes upon the lipid environmental changes in mammalian cells. Consistent with a recent in vitro solution NMR study on the pre-fusion state of VAMP2^27^, we observed a transient and dynamic membrane binding of VAMP2 with the affinity increasing from the N-terminal proline-rich domain to the C-terminal juxta-membrane domain. In the previous study, it was suggested that lipid binding of VAMP2 may generally lower the energy barrier such as to promote membrane fusion^27^. While, in this work, we find that different regions in the SV membrane can finely tune the conformations of VAMP2, which results in different activities of VAMP2 in the SNARE assembly (Fig. 5). As localizing on the cholesterol-rich lipid rafts, the SNARE motif of VAMP2 tends to be less associated with the membrane and more active to engage in the SNARE assembly together with syntaxin-1 and SNAP25 (Fig. 5)^28^. While, as localizing elsewhere on the SV membrane, the SNARE motif tends to be more associated with the membrane, likely in an α-helical conformation^10,11^, and less active for the SNARE assembly (Fig. 5). Thus, although the flexible binding of VAMP2 to membranes overall can facilitate the SNARE assembly in comparison to a tight binding, this process is under an exquisite regulation with different VAMP2 activity states by the uneven membrane compositions.

In this work, we quantitatively profiled the lipid compositions of lipid-raft and non-raft regions of SV membrane (Supplementary Data 1). We have shown the influence of the different electrostatic surfaces of these two regions on the binding affinities of VAMP2. It is known that polyphosphoinositides, such as PI-4,5-bisphosphate (PIP2), are key in regulating SV recycling^29^; however, their interactions with VAMP2 remain unknown. Our work showed that negatively charged PI exhibited a strong affinity to VAMP2 (Fig. 4a). PIP2, as a phosphorylated derivative of PI, contains more negative charges and thus is assumed to have stronger interaction with VAMP2. SM is known to be enriched in lipid raft fractions and usually associated with cholesterol^30^. Indeed, as we measured, SM in lipid raft and non-raft fractions are 0.75 ± 0.019 mol% and 0.12 ± 0.011 mol% (or 11.80 ± 0.144 pmol μg^−1^ protein and 0.74 ± 0.147 pmol μg^−1^ protein), respectively (Fig. 3a and Supplementary Data 1). Thus, we assume that SM species may also play a role in modulating VAMP2 membrane binding. Besides, we noticed differences between lipid rafts and non-rafts in the length and saturation of lipid aliphatic tails. Specifically, phospholipids with shorter tails are enriched in the lipid rafts (Supplementary Fig. 13a), while phospholipids with unsaturated tails are enriched in the non-raft membranes (Supplementary Fig. 13b). A previous study reported that high contents of unsaturated fatty acyl chains can help to support rapid vesicle fission because of its low energetic cost in membrane bending^31^. These data may also explain the phenomenon that cholesterol-depleted neurons, with a decrease of lipid rafts and an increase of unsaturated phospholipids, have an increased level of spontaneous fusion^32^.

Membrane microdomains have been suggested as functional loci of various proteins involved in different pathways^24^. Previous studies show that the SANRE proteins are highly enriched on lipid rafts in different cells^21,22^. Reducing the cellular cholesterol level to disrupt lipid rafts can impair the regulated exocytosis^22^. In addition, in the Niemann-Pick disease type C1, cholesterol is deficient to be recruited to neuronal membranes, which causes dramatic defects in evoked neurotransmission^33^. Despite the important role of lipid rafts, the in vitro studies have been debated, especially on the potential artifacts introduced during the extraction of lipid rafts^34^. No matter whether the extracted lipid rafts exactly represent the native counterpart, at least it is clear that lipids are sorted by different microdomains within the cell membranes^35^. Our work demonstrates that membrane microdomains not only recruit functional proteins, and also directly regulate protein structures to facilitate interactions. Both membrane lipids and proteins (e.g., syntaxin-1^36^ and synaptotagmin-1^37^) may cooperate to regulate VAMP2 structure, but lipid distribution and regionalization may add a spatial dimension to generally modulate SNARE protein conformations and SNARE complex assembly.

The extravesicular domain of VAMP2 features an intrinsically disordered sequence which is unstructured in the form of monomer. Proteins containing intrinsically disordered regions (IDRs) present as a heterogeneous ensemble, which may undergo distinct conformational changes in different biological contexts. As for VAMP2, we demonstrate that it transforms between different conformations as localizing on different regions of SV membranes and in complex with other core SNARE proteins. About 70% of human membrane proteins involved in signaling contain IDRs^38^. Many of them (e.g., T cell receptor-CD3^39^ and epidermal growth factor EGFR^40^) have multiple binding partners including proteins, lipids and metabolic ions. The technologies performed in this study may be useful for the structural study of other IDR-containing membrane proteins in live cells and physiological-relevant circumstances.

## Methods

### Chemical Standards of Lipids

The lipid chemical standards were purchased from Avanti Polar lipids: PC(15:0/18:1-d7) (791637), PE(15:0/18:1-d7) (791638), PG(15:0/18:1-d7) (791640), PS(15:0/18:1-d7) (791639), PI(15:0/18:1-d7) (791641), PA(15:0/18:1-d7) (791642), Ceramide(d18:1-d7/15:0) (860681), SM(d18:1/18:1-d9) (791649), LPC(18:1-d7/0:0) (791643), LPE(18:1-d7/0:0) (791644), TG(15:0/18:1-d7/15:0), Cholesterol-d7 (700041). Cholesterol(ovine) (700000), DOPC (850357), DOPE (850725), DOPS (840035), DOPG (840475), Brain PC (840053), Brain PE (840022), Brain PS (840032), Egg PG (841138), Liver PI (840042), Egg PA (840101), Biotinyl PE (870828).

### Protein purification

The rat VAMP2(1–96), VAMP2(1–78) and VAMP2(1–59) fused with His-tags were overexpressed from pET31b plasmids in *E. coli* BL21-DE3 (CodenPlus). Primer sequences used for making the two shorten constructs were: VAMP2(1–78)-F: GTTTG AATAA AGTGC AGCCA AGCTC AAGCG, VAMP2(1–78)-R: CTGCA CTTTA TTCAA ACTGG GAGGC; VAMP2(1–59)-F: CCAGA AGTAA TCGGA ACTGG ATGAT CGCGC AG, VAMP2(1–59)-R: GTTCC GATTA CTTCT GGTCT CGCTC C. Non-isotope enriched proteins were produced in LB medium, ^15^N-labeled proteins were produced in M9 minimal media supplemented with ^15^NH_4_Cl (1 g L^−1^, CIL). Bacteria were harvested by centrifugation after induction by 1 mM IPTG at 37 °C for 6 h in LB medium or 12 h in M9 media with the OD_600_ value of around 2.0. The bacteria were lysed by high pressure in a lysis buffer (50 mM Tris-HCl, pH 7.4, 100 mM NaCl, 1 mM PMSF).

Since the overexpressed VAMP2(1–96) forms inclusion bodies *in E.coli* the inclusion bodies in pellets were spun down (16,000 × *g* for 30 min) and washed twice in 10% Trinton-X100 and 1 M NaCl buffer (25 mM Tris-HCl, pH 7.4) to remove lipids, nucleotides and other proteins. Then the inclusion bodies were solubilized in 6 M guanidine hydrochloride buffer (25 mM Tris-HCl, pH 7.4) and purified by HisTrap™ HP columns (GE Healthcare). The purified protein was harvested in 6 M guanidine hydrochloride buffer (25 mM sodium phosphate, pH 2.0) and further purified by RP-HPLC C3 column (Agilent Technology). The lyophilized protein was solubilized and cleaved by TEV protease at 4 °C overnight in buffer (50 mM Tris-HCl, pH 7.4, 100 mM NaCl, 1 mM DTT) to remove His-tag. Finally, the VAMP2(1–96) proteins without His-tag was purified by RP-HPLC C8 column and lyophilized.

VAMP2(1–78) and VAMP2(1–59) were overexpressed in *E.coli* as soluble proteins in the supernatants (after centrifugation at 16,000 × *g* for 30 min) and were purified by HisTrap^TM^ HP columns. Then, the His-tag proteins were dialyzed into the cleavage buffer as above and cleaved by TEV protease at 4 °C overnight. At last, the enzyme-digested VAMP2 proteins were purified by RP-HPLC C8 column and lyophilized.

The rat full-length VAMP2, syntaxin-1a and SNAP25 were gifted from lab of Jinshi Shen (Colorado, USA). The three proteins were purified as reported^41^. The human α-synuclein protein was purified by following the protocol published^42^.

### Electroporation of purified proteins into mammalian cells

Human HEK-293T (ATCC, CRL-3216) and SH-SY5Y (ATCC, CRL-2266) cells were cultured following the protocol provided by ATCC. Both cell lines were tested for mycoplasma contaminations and were mycoplasma free. The cells with four to six passage were used for NMR experiments.

The purified protein powder of VAMP2(1–96), VAMP2(1–78), VAMP2(1–59) or α-synuclein was dissolved in Buffer R supplied in the Neon transfection system kit (Invitrogen, MPK10025) to a final concentration of 300 μM. Cells were collected by trypsinization and washed with PBS for three times to remove the culture medium. Then the cells were resuspended with VAMP2 solution with the density of 8 × 10^7^ cells mL^−1^ for HEK-293T and 4 × 10^7^ cells mL^−1^ for SH-SY5Y. Electroporation was conducted using 100 μL of the cells mixed with protein with a pulse program of 1400 V (pulse voltage), 20 ms (pulse width) and 2 pulses by the Neon transfection system (Invitrogen, MPK5000). The control sample was conducted with the identical setup but without the electric shocks.

For immunofluorescence and time-course immunoblotting experiments, aliquots of 0.5 × 10^6^ cells were added to each well in a 24-well plate, filled with 0.5 mL medium. For in-cell NMR and sub-cellular fractionation experiments, aliquots of 4–8 × 10^6^ cells were added to eight 10-cm dishes with 10 mL medium and cultured for 3~6 h for cell recovery. Then the cells were harvested and washed with PBS for four times. The in-cell NMR sample in 160 μL pH-stable L-15 medium (Gibco, 11415064) and 40 μL D_2_O was settled into the NMR tube by gentile sedimentation with a hand-cranked centrifuge. The suspended cells and additional medium were discarded. Finally, 500 μL sedimented cell slurry was prepared for NMR measurement. The cell sample for sub-cellular fractionation were stored at −80 °C prior to the experiments.

To determine the intracellular concentration of VAMP2(1–96), and check its potential cell leakage after in-cell NMR experiments, the cell samples were centrifugated at 300 × *g* for 3 min. Then the supernatants (S) and pellets (P) were resuspended in Laemmli buffer to volume of 500 μL, and boiled for 10 min. As for the immunoblotting experiment, the samples were diluted by 10-times and 10 μL sample was loaded in each lane of a 15% SDS-PAGE gel. Uncropped and unprocessed scans of all blots were in Source Data file. The concentration of the electroporated VAMP2(1–96) in-HEK-293T-cell (~10 μM) and in-SH-SY5Y-cell (~80 μM) samples were analyzed and calculated by using ImageJ^43^ (Fig. 1c).

### Sub-cellular fractionation of the electroporated cells

Isolation of the cytosol and membrane fractions of the cells was achieved by following a published protocol^44^. The experiment was performed at 4 °C or on ice with pre-cooled reagents. Briefly, aliquots of 2–4 × 10^7^ electroporated cells were permeabilized by adding 2 mL of digitonin lysis buffer (110 mM KOAc, 2 mM MgCl_2_, 20 mM K-HEPES, pH 7.5, 0.02% digitonin) with protease inhibitors for 10 min to release cytosolic contents. The lysate (Lys) was centrifuged at 20,000 × *g* for 10 min. The supernatant was the cytosol fraction (Cyto). The permeabilized cell pellet was washed three times by the lysis buffer and centrifuged at 20,000 × *g* for 10 min. The pellet was resuspended in 2 mL lysis buffer as for the membrane fractions (Mem).

The Lys, Cyto and Mem samples were further characterized by immunoblotting. The antibodies used include VAMP2 (SYSY, 104211, 1:10,000), GAPDH (Cell Signaling Technology, 2118, 1:1000) for cytosol, IRE1α (Cell Signaling Technology, 14C10, 1:1000) for endoplasmic reticulum, ACSL4 (Santa Cruz, sc-365230, 1:1000) for plasma membrane-associated membranes, and VDAC (Cell Signaling Technology, 4661, 1:1000) for mitochondria. Uncropped and unprocessed scans of all blots were in Source Data file. Immunoblotting quantification (Fig. 1d, and Supplementary Fig. 4a) of the proportion of electroporated protein in cytosol (Cyto/(Cyto+Mem)) was analyzed by ImageJ^43^.

### Immunofluorescence staining of the cultured cells

For immunofluorescence imaging, cells with or without the electroporated proteins were recovered for 3~6 h on poly-D-lysine-coated coverslips in 24-well plates. Then, the cells were washed by pre-warmed PBS three times to remove extracellular un-delivered proteins, then fixed in 4% (w/v) paraformaldehyde in PBS for 30 min and permeabilized with 0.1% (v/v) Triton X-100 in PBS for 20 min. After washing with PBS three times, cells were blocked with 10% BSA in PBS for 1 h followed by incubation of antibodies at 4 °C overnight, including Oyster-550 labeled VAMP2 (SYSY, 104211C3, 1: 1000), α-synuclein (BD Biosciences, 610787, 1: 1000) and FITC-labeled phalloidin (Yeasen, 40736ES75, 1:200) for F-actin filaments beneath cell membranes^45^. Then Alexa Fluor 594-conjugated secondary antibodies (Invitrogen, A-11020, 1:500) were used. Slides were then washed with PBS for three times. The nucleus was stained by antifade mountant coupled DAPI (Invitrogen, P36935). Finally, the samples were observed by a confocal microscope (Lecia, SP8).

### In-cell and in vitro solution NMR spectroscopy

All the NMR experiments were carried out at 25 °C on a Bruker 900 MHz spectrometer equipped with a cryogenic probe. The buffer used in all of the in vitro NMR experiment was 50 mM sodium phosphate buffer (pH 6.5) containing 50 mM NaCl and 10% D_2_O (v/v). For the in vitro titration experiments, liposomes were prepared with a concentration of 50 mM, and were gradually added into the solution containing 25 μM ^15^N-VAMP2(1–96) to the indicated molar ratios with a final volume of 500 μL for NMR measurement. As for the SV titration experiment, the concentration of SV was calculated according to the ratio of its total proteins and phospholipid which was used (*25*). Bruker standard SOFAST-HMQC pulse sequence^46,47^ was used and the ^1^H shape pulse efficient was optimized for collecting the 2D NMR spectrum of the in-cell samples with 80 scans. The delay time (D1) was set to 0.29 s, and 1024 and 128 complex points were used for ^1^H and ^15^N, respectively. The experiment duration time is 61 min 16 s. Cell viability before and after the SOFAST-HMQC NMR experiment was assessed by trypan blue staining. Cell viability remained above 90% after the NMR experiments (Supplementary Fig. 14a), and damaged cells ranged from 2% before the experiments to 8% after the experiments on average.

Bruker standard pulse program hsqct1etf3gpsi and hsqct2etf3gpsi were used as described by Farrow et al.^48^ and Liu et al.^49^ to measure the backbone ^15^N relaxation parameters of R_1_ and R_2_ from the VAMP2 electroporated HEK-293T and SH-SY5Y cell samples as well as 50 μM VAMP2(1–96) in-solution protein sample, respectively. For in-solution NMR samples, the time delays for R_1_ experiment were 10, 40, 100, 200, 300, 500, 700, 800, 1200, and 2000 ms, while those for R_2_ experiments were 0, 20, 40, 80, 120, 200, and 400 ms, and the number of scan were 16. For in-cell NMR samples, the time delays for R_1_ experiment were 10, 40, 100, 200, 300, 500, 700, 900, and 1200 ms, while those for R_2_ experiments were 0, 20, 50, 80, 120, 200, and 400 ms, and the number of scan were 32. R_1_ and R_2_ relaxation experiments for in-cell samples took about 23 and 22 h, respectively. Cell viability of HEK-293T and SH-SY5Y cells remained 70%-80% after the hours-long NMR relaxation experiments (Supplementary Fig. 14b). Note that the lower viability could affect the R_1_ and R_2_ measurements due to protein leakage.

Backbone resonance assignment of VAMP2(1–96) was accomplished according to the Biological Magnetic Resonance Bank (BMRB) entry 4272 ^8^. Residue T27, S61 and eight prolines in the N-terminal of VAMP2 cannot be assigned. All of the NMR data were processed by NMRpipe^50^ and analyzed by SPARKY^51^. Specifically, the R_1_ and R_2_ relaxation data were analyzed using SPAKY relaxation fitting extension. The Residue-resolved relative signal intensity ratios (*Y*) of in-cell (*I*) to in-solution (*I*_0_) NMR spectrum were calculated for each residue *X* as following equation:

1 $$Y=[I(X)/I_0(X)]/[I(5)/I_0(5)]$$

where the intensity ratio of the residue *X* was normalized by the highly flexible residue 5 for comparing the regional flexibility of VAMP2(1–96) in cells (Fig. 1g). The residue-resolved relative NMR signal changes (Z) of VAMP2(1–96) in different treated cells (*Y*) comparing to it in untreated control cells (*Y*_0_) were calculated as following equation (Fig. 2c):

2 $$Z(X) = (Y-Y_0)/Y_0$$

### Manipulation of the cellular cholesterol level

Manipulation of the cholesterol level within cells was achieved by previous protocols^23^. In brief, to elevate the cellular cholesterol level, cells were treated with 20 μg mL^−1^ Cholesterol-MβCD (Sigma, C4951) for 8 h before the electroporation experiment and lipidomic profiling. To decrease the cellular cholesterol level, the cell cultured medium was replaced to lipoprotein deficient serum (LPDS) supplemented medium containing 5 μM mevastatin and 50 μM mevalonate (Sigma, M2537 & 90469) and the cells were further cultured for 8 h. Then the cells were treated with 5 mM MβCD for 20 min prior to the electroporation experiment and lipidomic profiling. The LPDS was gifted from lab of Chenqi Xu (Shanghai Institutes for Biological Sciences, CAS.), which was prepared as published protocol^23^. The total cellular cholesterol level was quantified using the Amplex Red Cholesterol Assay Kit (Invitrogen, A12216), and normalized by the total cellular protein concentration measured by a Pierce BCA Protein Assay Kit (Thermo Fisher Scientific, 23225). The cholesterol level in HEK-293T cells was able to be up- or down-regulated by ~60%, respectively; the cholesterol level in SH-SY5Y cells was not able to be downregulated.

### Isolation of synaptic vesicles from mouse brains

Synaptic vesicles (SV) were purified following a published protocol^52^. The experiment was performed at 4 °C or on ice with pre-cooled reagents. Four brains from 8-week-old C57BL6 mice (male) were homogenized (900 r.p.m. for 10 min) in 25 mL 4 mM Na-HEPES, pH 7.4 and 320 mM sucrose buffer (HB) with protease inhibitors by using a PTFE pestle in a 40 mL glass tube (Sigma, P7984). The homogenate was centrifuged at 1500 × *g* for 10 min. The supernatant (S1) was collected and kept on ice. The pellet was resuspended with 25 mL HB and homogenized (900 r.p.m. for 10 min), followed by centrifugation at 1500 × *g* for 10 min. Then the supernatant (S2) combined with S1 were centrifuged at 20,000 × *g* for 20 min. The pellet which contains synaptosomes was resuspended with 2 mL HB and then homogenized in 20 mL H_2_O at 1200 r.p.m. for 10 min followed by adding 50 μL of 1 M Na-HEPES, pH 7.4 and protease inhibitors. The homogenate was placed on ice for 30 min, and centrifuged at 20,000 × *g* for 20 min. The supernatant was ultra-centrifuged at 70,000 × *g* for 45 min. The pellet was clustered SV extracts. The samples for different fraction were diluted with a total protein concentration of 50 μg mL^−1^ for immunoblotting. Antibodies used included synaptophysin (Sigma, S5768, 1:1000) for SVs, VDAC for mitochondria, IRE1α for endoplasmic reticulum and GAPDH for cytoplasm. Uncropped and unprocessed scans of all blots were in Source Data file. To obtain homogenous SVs, fraction SV was resuspended in NMR buffer and homogenized by using a PTFE pestle in a 3 mL glass tube (Sigma, P7734) at 1200 r.p.m. for 10 min. Furthermore, to disrupt any remaining SV clusters before NMR experiment, the homogenized SVs was drawn through a 20-gauge hypodermic needle attached to a 10-mL syringe, and then changed to a 27-gauge needle and expelled.

### Isolation of lipid-raft and non-raft membranes from SVs

Fractionation of lipid-raft and non-raft membranes from SVs was achieved by using a published protocol^53^. Briefly, the isolated SV was suspended with 4 mL ice-cold Mes-buffer (25 mM Mes, pH 6.5, 150 mM NaCl, phosphatase inhibitors) supplemented with Triton X-100 to a final concentration of 1% v/v. The mixture was gently swung at 4 °C for 10 min, and then homogenized using a PTFE pestle in an 8 mL glass tube (Sigma, P7859) at 200 r.p.m. for 10 min, followed by adding 80% (w/v) sucrose in Mes-buffer with a final concentration of sucrose of 40% (w/v). The mixture was divided equally into two 13.5 mL ultracentrifuge tubes (Beckman, 344059) and overlaid successively with 6 mL 30% (w/v) sucrose and 2.5 mL 5% (w/v) sucrose. After centrifugation (Beckman, SW41Ti rotor) at 240,000 × *g* for 6 h, 12 fractions (1 mL per fraction) were collected from the top to the bottom. The pellet was resuspended in Mes-buffer to a total volume of 1 mL as fraction 13. The different fractions were analyzed by immunoblotting. Antibodies used in the experiment included flotillin2 (Santa Cruz, sc-28320, 1:500) for lipid rafts, rabphilin3A (Santa Cruz, sc-393197, 1:500) for detergent-soluble non-raft membranes and VAMP2. Uncropped and unprocessed scans of all blots were in Source Data file. By analysis of the immunoblot, the fraction 3 was stored as the lipid-raft sample and fraction 9–12 were mixed stored as the non-raft sample.

### Lipid extraction from biological samples

The lipids in lipid-raft and non-raft fractions were extracted using a modified MTBE extraction method. 100 μL of each lipid-raft was mixed with 100 μL H_2_O and 480 μL of extraction solvent (MTBE: MeOH = 5:1, v/v, containing 1.5 μg of PC(15:0/18:1-d7), 0.5 μg of PE(15:0/18:1-d7), 0.25 μg of PG(15:0/18:1-d7), 0.5 μg of PS(15:0/18:1-d7), 0.5 μg of PI(15:0/18:1-d7), 0.05 μg of PA(15:0/18:1-d7), 0.2 μg of ceramide(d18:1-d7/15:0), 0.05 μg of SM(d18:1/18:1-d9), 0.02 μg of LPC(18:1-d7/0:0), 0.02 μg of LPE(18:1-d7/0:0), 10 μg of TG(15:0/18:1-d7/15:0)). 100 μL of each non-raft sample was mixed with 100 μL H_2_O and 480 μL of extraction solvent (MTBE: MeOH = 5:1, v/v, containing 1.5 μg of PC(15:0/18:1-d7), 0.5 μg of PE(15:0/18:1-d7), 0.25 of μg PG(15:0/18:1-d7), 0.5 μg of PS(15:0/18:1-d7), 0.5 μg of PI(15:0/18:1-d7), 0.05 μg of PA(15:0/18:1-d7), 0.02 μg of ceramide(d18:1-d7/15:0), 0.01 μg of SM(d18:1/18:1-d9), 0.02 μg of LPC(18:1-d7/0:0), 0.02 μg of LPE(18:1-d7/0:0), 10 μg of TG(15:0/18:1-d7/15:0)). Each sample was vortexed for 30 s, followed by 10 min of sonication and 15 min of centrifugation at 13,000 × *g*. The upper organic layer was collected. Then, 200 μL of MTBE was added to the left aqueous layer for re-extraction. The re-extraction process was repeated twice, and the pooled organic layer was evaporated using a vacuum concentrator. The dried extract was reconstituted in 200 μL of DCM: MeOH (1:1, v/v) for lipid quantitative analysis.

For the quantification of cholesterol, 20 μL of each lipid-raft or non-raft sample was firstly diluted to 200 μL using H_2_O. Then 100 μL of each sample was mixed with 100 μL H_2_O and 480 μL extraction solvent (MTBE: MeOH = 5:1, v/v, containing 2 μg of cholesterol-d7). Each sample was vortexed for 30 s, followed by 10 min of sonication and 15 min of centrifugation at 13,000 × *g*. The upper organic layer was collected. Then, 200 μL of MTBE was added to the left aqueous layer for re-extraction. The re-extraction process was repeated twice, and the pooled organic layer was evaporated using a vacuum concentrator. The dried extract was reconstituted in 100 μL of DCM: MeOH (1:1, v/v) for LC-MS analysis. An external calibration curve of cholesterol was also measured with a linear range from 0.5 μg mL^−1^ to 200 μg mL^−1^. The internal standard cholesterol-d7 (20 μg mL^−1^) was added into each calibration sample. Finally, the absolute concentrations of cholesterol in lipid-raft or non-raft samples were quantified according to the measured peak area of cholesterol in comparison to the internal standard (cholesterol-d7) by interpolation from the calibration curve.

For the quantification of cholesterol in HEK-293T cells, 20 μL of each cell sample was firstly diluted to 200 μL by using H_2_O. Then, 100 μL of each sample was mixed with 100 μL of H_2_O and 480 μL of extraction solvent (MTBE: MeOH = 5:1, v/v, containing 1 μg of cholesterol-d7). The rest procedures were the same as the preparation of lipid-raft samples. Finally, the dried extract was reconstituted in 100 μL of DCM: MeOH (1:1, v/v). A calibration curve of cholesterol was also measured with a linear range of cholesterol from 1 μg mL^−1^ to 100 μg mL^−1^, and cholesterol-d7 (10 μg mL^−1^) was also added into each calibration sample as internal standard. The total protein concentration of each cell sample was measured at beginning by a Pierce BCA Protein Assay Kit (Thermo Fisher Scientific, 23225). The cellular cholesterol concentration was normalized to the corresponding protein concentration of each sample.

### LC-MS based lipidomics

The LC-MS based lipidomic analyses were performed by using a UHPLC system (Agilent Technologies, 1290 series) coupled to a quadrupole time-of-flight mass spectrometer (Sciex, TripleTOF 6600). Chromatographic separations were performed on a Phenomenex Kinetex C18 column (particle size, 1.7 μm; 100 mm (length) × 2.1 mm (i.d.)) with column temperature kept at 55 °C. The mobile phases A = 10 mM ammonium formate in H_2_O: ACN (6:4, v/v), and B = 10 mM ammonium formate in IPA: ACN (9:1, v/v), were used for both ESI positive and negative modes. The linear gradient elutes from 40 to 100% B (0–12 min), 100% B (12–14 min), 100 to 40% B (14–14.2 min), then equilibrate at 40% B until 18 min. The flow rate was set as 0.3 mL min^−1^. The mass spectrometry parameters were applied as follows: ion source gas 1 (GS1), 60 psi; ion source gas 2 (GS2), 60 psi; curtain gas (CUR), 30 psi; temperature, 600 °C; ion-spray voltage floating (ISVF), 5000 V or -4500 V in positive or negative modes, respectively; de-clustering potential (DP), 100 V. Data-dependent acquisition (DDA) method was used for MS/MS acquisition. Each acquisition cycle consists of one rapid TOF MS survey scan (200 ms) followed by the consecutive acquisition of 11 product ions scans (50 ms each). The collision energy (CE) was set as 45 V, and CE spread was set as 15 V. At the beginning of each data acquisition batch, a commercially available lipid mixture (Avanti Polar Lipids, 330708) was first analyzed, and used for retention time correction and re-calibration. Please refer to our recent publications for detailed protocols^54,55^.

### Lipid identification and quantification

LC-MS raw data (.wiff) files were converted to the mzXML format using ProteoWizard (version 3.0.6150), and processed by LipidAnlayzer developed in our lab^54^. First, peak detection and alignment were performed using the CentWave algorithm and the ordered bijective interpolated warping (OBI-Warp) algorithm in XCMS. Then, lipid identification was achieved through the combination of accurate mass, MS/MS spectral similarity match, and retention time (RT) match. An in-silico MS/MS spectral database modified from LipidBlast^56^ was developed and reported in our recent publications^54,57^. An accurate mass match was firstly performed to search all molecular species in the database. For each matched molecular species, the corresponding experimental MS/MS spectrum was further matched to the MS/MS spectral database. Here, a reverse dot-product function^58^ was used to evaluate the MS/MS spectral similarity.

3 $${\mathrm{Spectral}}\;{\mathrm{similarity}}\;{\mathrm{score = }}\frac{{{\sum} {\left( {(\left[ I \right]_D^n\left[ {{\mathrm{mz}}} \right]_D^m{\mathrm{)(}}\left[ I \right]_E^n\left[ {{\mathrm{mz}}} \right]_E^m)} \right)} }}{{{\sqrt {{\sum} {\left( {(\left[ I \right]_D^n\left[ {{\mathrm{mz}}} \right]_D^m)^2{\sum} {(\left[ I \right]_E^n\left[ {{\mathrm{mz}}} \right]_E^m)^2} } \right)} } } }}$$

where mz and *I* refer to the mass-to-charge value and intensity from the database (*D*) or experimental (*E*) data, respectively, while *m* and *n* represent the weight of mz and intensity, respectively. Here, we set *m* = 1 and *n* = 0.6 (positive mode) or 1 (negative mode), which was systematically optimized for lipid identification in our system^54^. The similarity score ranged from 0 to 1, referring to no similarity and a perfect match, respectively. Lipid matches with scores larger than 0.8 were kept as candidates.

In addition, experimental RTs of lipids were also matched with RTs in the database to filter the false positive identifications. Please refer to our recent publication for detailed protocol^55^. Briefly, a predicted RT database was developed using random forest based machine-learning algorithm^55^. A commercial lipid mixture (Avanti Polar Lipids, 330708) was used for RT calibration. Next, a trapezoidal function was used to score the RT similarity for each candidate. The RT score ranges from 0 to 1, referring to no match and a perfect match, respectively. We only kept lipid identifications with MS/MS spectral similarity score larger than 0.8 and RT similarity score larger than 0.5.

Finally, the deuterated internal standards were used to quantify the corresponding lipid species within the linear range. In this experiment, a total of 12 deuterium labeled internal standards were added to samples before extraction, and the lipid species were quantified by measuring areas under curve (called peak area) in comparison to the corresponding internal standards, and then multiplying the amount of the internal standard. If one lipid with the concentration exceeding up-limit of the linear range, it was re-analyzed after dilution.

For comparing the lipid subclass between lipid raft and non-raft membrane fractions, we first summed up all individual molecular species (noted in Supplementary Data 1) in each lipid subclass and then normalized by the sum of all lipids in each sample.

### Liposomes and vesicles reconstituted with SNAREs

The different lipid molecules for liposome preparation used in this study were dissolved and mixed in chloroform and evaporated using a dry nitrogen stream. The dried lipid film was hydrated by adding the NMR buffer or HEPES buffer (100 mM NaCl, 25 mM HEPES, pH 7.4), and then ultrasonicated in a water bath at 65 °C for 10 min. To prepare liposomes with homogenous size, the hydrated lipids were extruded 21 times at 65 °C through a polycarbonate film with a pore size of 50 nm (Whatman Nucleopore Track-Etch) by using an extruder apparatus (Avanti Polar Lipids, 610000). The size and homogeneity of the liposomes were confirmed by dynamic light scattering instrument (Wyatt Technology, 431-DPN).

In single-vesicle docking experiment, to prepare the SNARE protein reconstituted labeled vesicles, 2 mol% of brain PC and 0.5 mol% of brain PE of the lipid-raft and non-raft vesicles were substituted by 2 mol% DiI or DiD (Invitrogen, D282 or D307) and 0.5 mol% biotinyl PE. Then, DiI labeled vesicles and full-length VAMP2 were mixed and incubated for 30 min on ice. DiD labeled vesicles were added to pre-mixed syntaxin-1a and SNAP25 solution. Both of the mixtures were diluted with the same volume of HEPES buffer and dialysis in 2 L HEPES buffer at 4 °C overnight, the reconstituted vesicles were transferred into tubes for further research. The lipid-to-protein molar ratio was 200:1 based on VAMP2 or syntaxin-1a.

### Single-vesicle docking experiments

The prepared DiI and DiD vesicles reconstituted with neuronal SNARE proteins were used for single-vesicle docking experiments. The lipid to protein ratio was 200:1. In brief, the DiD t-vesicles reconstituted with syntaxin-1a & SNAP25 were firstly immobilized on the imaging surface of PEGylated quartz slides via a biotin/NeutrAvidin interaction. The DiD v-vesicle coverage was confirmed by a red laser excitation. After buffer change, DiI vesicles harboring full-length VAMP2 were injected into the sample channel and incubated for 30 min to be bound to the surface of immobilized t-SNARE vesicles. Before imaging under a wide-field total internal reflection fluorescence (TIRF) microscopy, the channels were washed by HEPES buffer three times to remove uncombined vesicles.

The smCamera program was used to acquire and analyze the images respectively. The number of vesicles docking was determined via counting the number of fluorescent spots of acceptor channel under green laser excitation (532 nm). 20 random locations were imaged and analyzed for each sample channel on the slide. The compared results were expressed as mean ± standard deviations. One-way analysis of variance (ANOVA) with Tukey Test was used to determine the statistical significance among different groups. When *P* < 0.001, the statistics were considered extremely significant (***).

### Reporting summary

Further information on research design is available in the Nature Research Reporting Summary linked to this article.

## Supplementary information

Supplementary Information

Supplementary Data 1

Reporting Summary

Description of Additional Supplementary Files

## Data Availability

Data supporting the findings of this manuscript are available from the corresponding author upon reasonable request. A reporting summary for this Article is available as a Supplementary Information file. The source data underlying Figs. 1c, d, g, h, 2c, 3b–d, f, 4a–c, and Supplementary Figs. 1, 3, 4, 5, 7, 8, 9a, b and 13a, b are provided as a Source Data file. The chemical shifts of VAMP2(1–96) in HEK-293T and SH-SY5Y cells were deposited in Biological Magnetic Resonance Bank (BMRB) under accession number 50199 and 50198, respectively. The lipidomics raw data of lipid raft and non-raft samples were deposited in MetaboLights under accession number MTBLS1503.

## Source Data

Source Data
