# Supplementary material for: Different regions of synaptic vesicle membrane regulate VAMP2 conformation for the SNARE assembly
Source: Nat Commun. 2020 Mar 24;11:1531. doi: 10.1038/s41467-020-15270-4 (PMC7093461; doi:10.1038/s41467-020-15270-4)
Supplement: Supplementary file 1 — Supplementary Information [file 41467_2020_15270_MOESM1_ESM.pdf]

Supplementary Information for

Different regions of synaptic vesicle membrane regulate VAMP2 conformation for the SNARE assembly

Wang *et al.*[1]

This file includes: Supplementary Figures 1 - 14

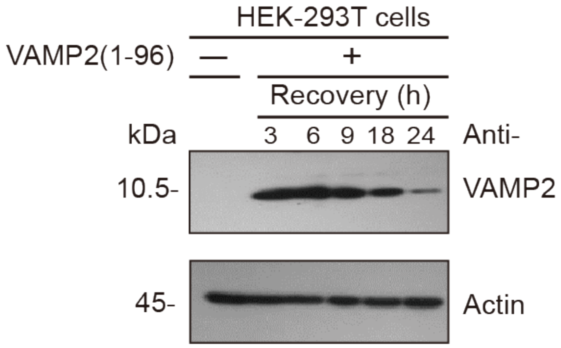

Supplementary Figure 1 | Half-life of delivered VAMP2 in cells. The relative amounts of VAMP2(1-96) in cells were measured by immunoblotting at different recovering time. Source data are provided as a Source Data file.

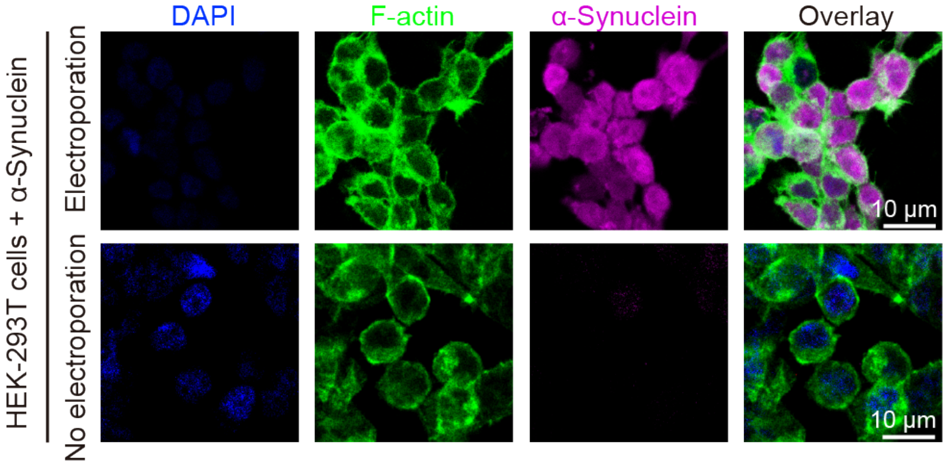

Supplementary Figure 2 | Distribution of delivered α-synuclein in cells. Cellular localization of α-synuclein was visualized by immunofluorescence staining. F-actin filaments depict cell skeletons stained by FITC-phalloidin and nuclei were stained by DAPI.

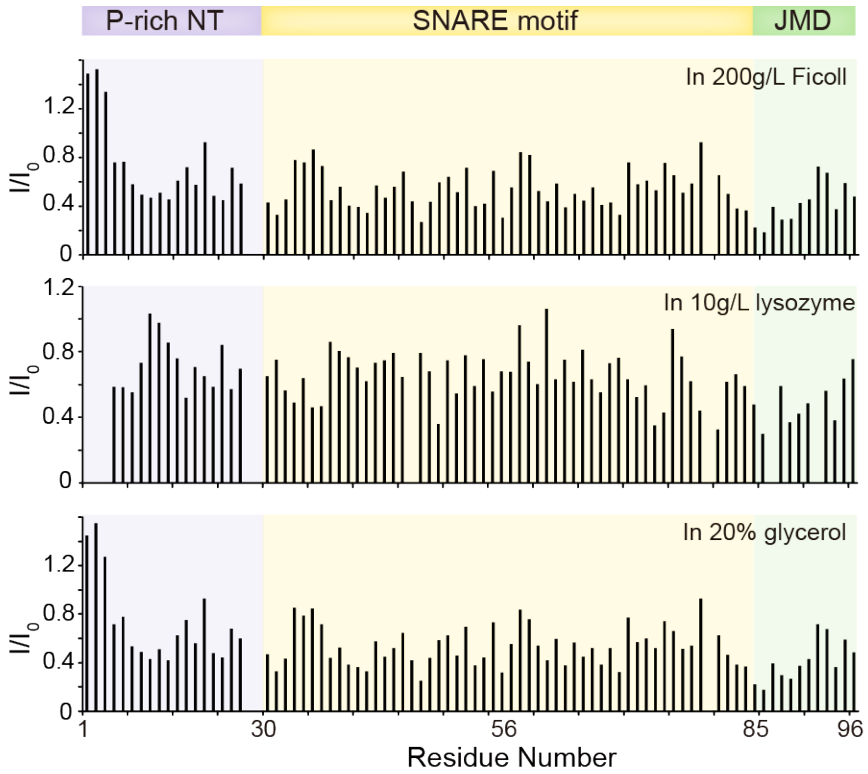

Supplementary Figure 3 | NMR titration of VAMP2 with different crowding agents. The concentration of VAMP2(1-96) is 25 μM. Concentrations of the crowding agents are indicated. Source data are provided as a Source Data file.

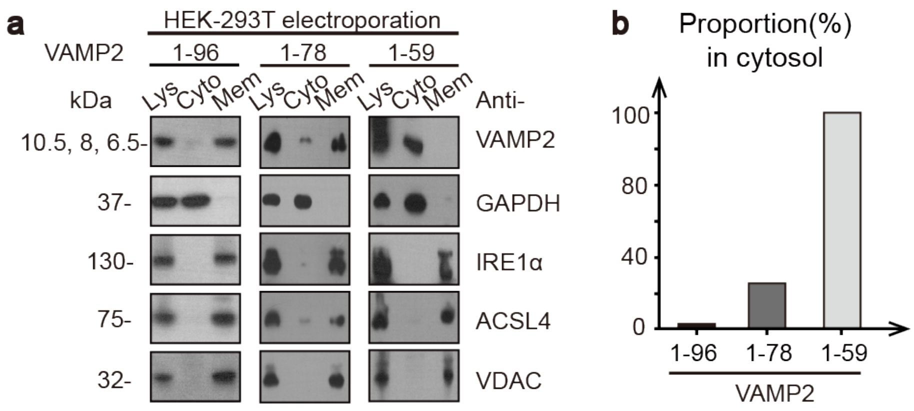

Supplementary Figure 4 | Sub-cellular localization of VAMP2 variants. a, Localization of delivered VAMP2(1-96), VAMP2(1-78) and VAMP2(1-59) in HEK-293T cells by immunoblotting. Fractions of the total lysates (Lys), cytosol (Cyto) and membrane (Mem) were validated by immunoblotting with antibodies noted in Fig. 1d. The immunoblotting of VAMP2(1-96) is the same as the right panel in Fig. 1d. **b**, Quantification of different VAMP2 variants in cytosol based on (**a**). Detailed information of data processing was noted in the Methods section. Source data are provided as a Source Data file.

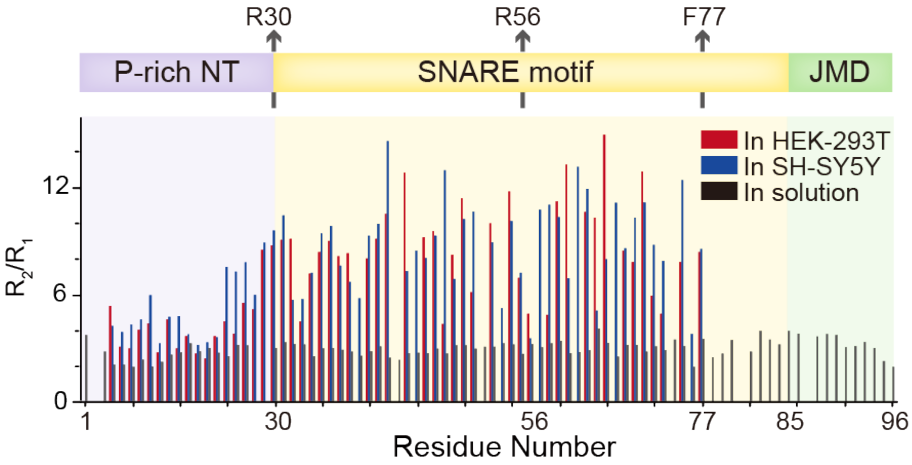

Supplementary Figure 5 | Residue-resolved ratios of ^{15}N transverse (R_2, s^{-1}) to longitudinal (R_1, s^{-1}) relaxation rates of VAMP2(1-96) in HEK-293T cells (red), SH-SY5Y cells (blue) and in solution (black). Source data are provided as a Source Data file.

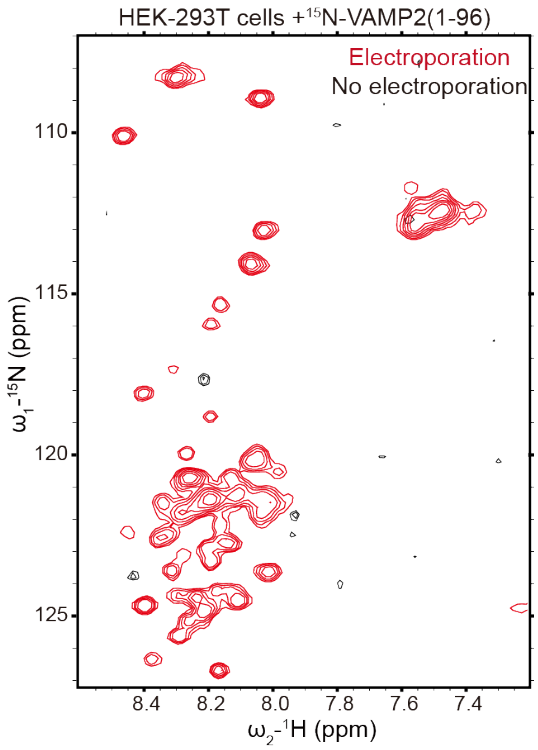

Supplementary Figure 6 | VAMP2 remainder on cell outer surface detected by NMR spectroscopy. HEK-293T cells were treated with VAMP2(1-96) in the same way as the sample preparation for in-cell NMR except for electroporation.

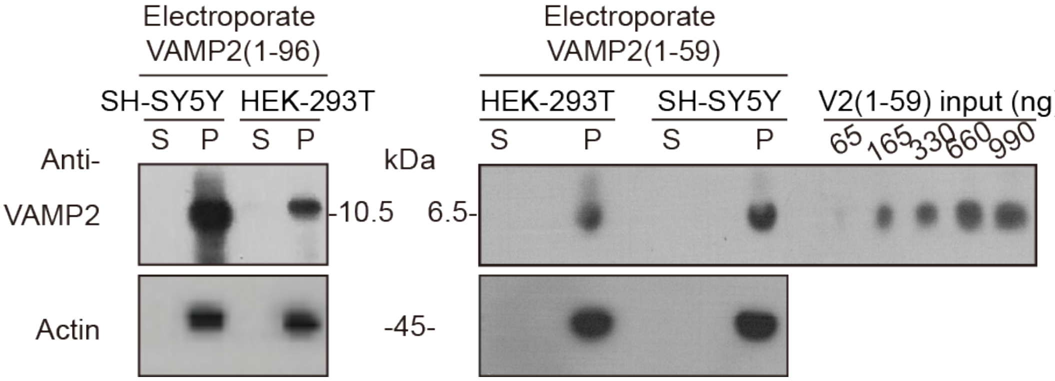

Supplementary Figure 7 | Leakage test for VAMP2 after the in-cell NMR experiments. The supernatant medium (S) and cell pellet (P) were collected for checking the leakage of VAMP2(1-96) and VAMP2(1-59) from cells over NMR measurements by immunoblotting. Source data are provided as a Source Data file.

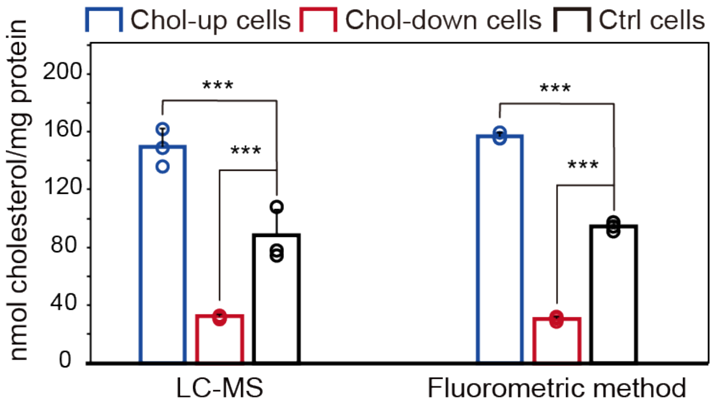

Supplementary Figure 8 | Quantification of cholesterol in cholesterol-regulated cells measured by LC-MS (left) and the fluorometric method (right). Error bars are standard deviations of three replicates. ***, p-value < 0.001; p-values were analyzed by Student's t-test. Source data are provided as a Source Data file.

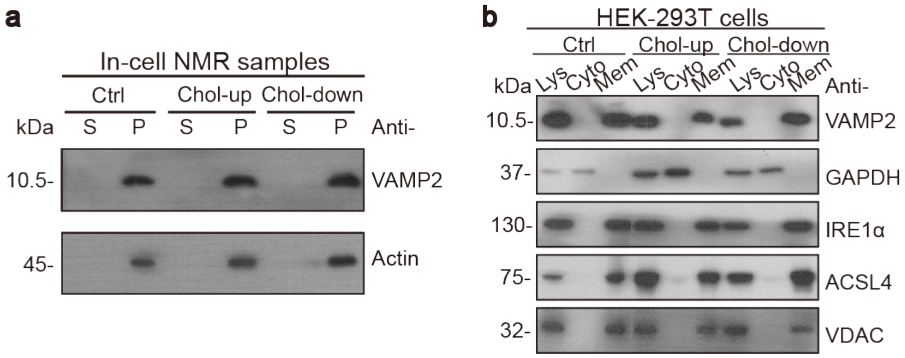

Supplementary Figure 9 | Amounts and localization of delivered VAMP2 in cholesterol-regulated cells. a, Comparisons of the leakage of VAMP2(1-96) in Chol-up, Chol-down and control HEK-293T cells by immunoblotting. The supernatant medium (S) and cell pellets (P) were collected after in-cell NMR experiments. **b**, Localization of VAMP2(1-96) in untreated control cells, Chol-up, and Chol-down regulated cells. Fractions of the total lysates (Lys), cytosol (Cyto) and membrane (Mem) were validated by immunoblotting with antibodies noted in Fig. 1d. Source data are provided as a Source Data file.

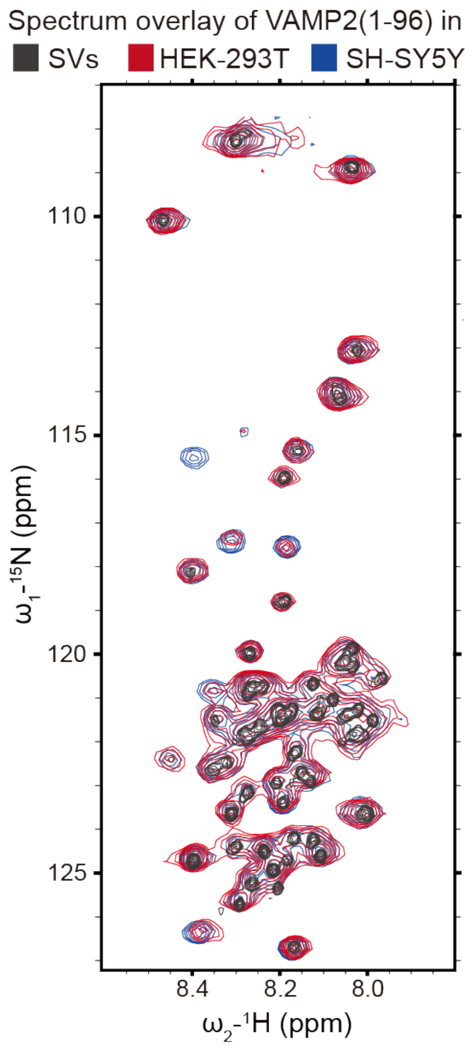

Supplementary Figure 10 | Overly of the 2D ^{1}H-^{15}N NMR spectra of VAMP2(1-96) with SVs (molar ratio is 700: 12), in HEK-293T cells and in SH-SY5Y cells.

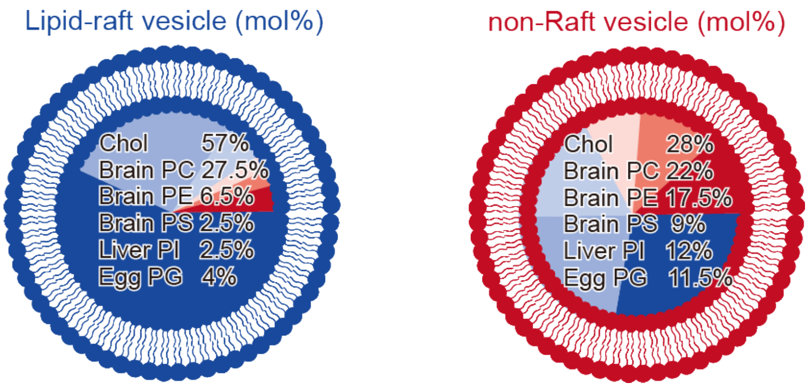

Supplementary Figure 11 | Lipid compositions of reconstituted lipid-raft- and non-raft-mimicking vesicles.

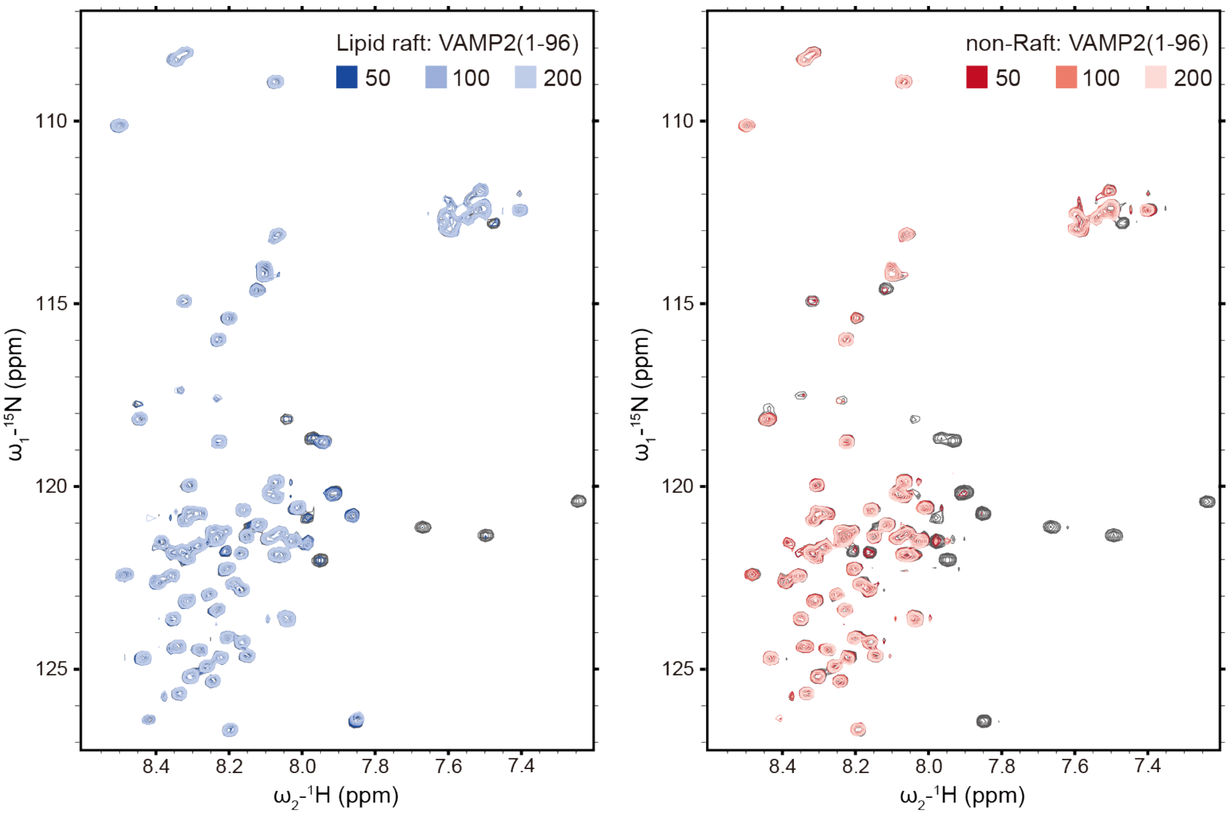

Supplementary Figure 12 | NMR titration of VAMP2 by lipid-raft- and non-raft-mimicking vesicles. 2D ^{1}H-^{15}N NMR spectra of VAMP2(1-96) are shown. The molar ratios of lipid to VAMP2(1-96) are indicated.

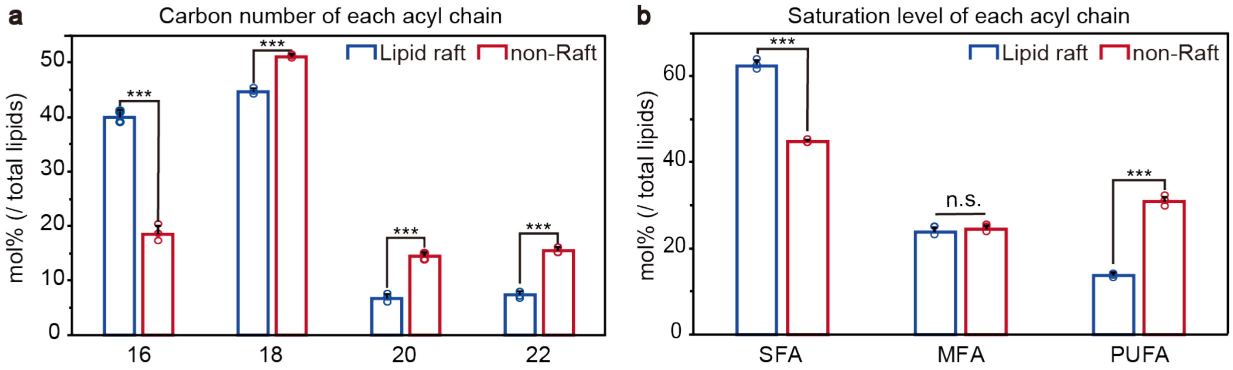

Supplementary Figure 13 | Comparisons of the acyl hydrocarbon chains of lipids in lipid-raft and non-raft membranes. a, The lipids with acyl hydrocarbon chains were categorized by length into four subclasses (carbon numbers are indicated). **b**, The acyl hydrocarbon chains were categorized by saturation levels into three subclasses. S: saturated; M: monounsaturated; PU: polyunsaturated. Error bars are standard deviations from three biological replicates. ***, *p*-value < 0.001; n.s. represents not significant. *p*-values were analyzed by Student's t-test. Source data are provided as a Source Data file.

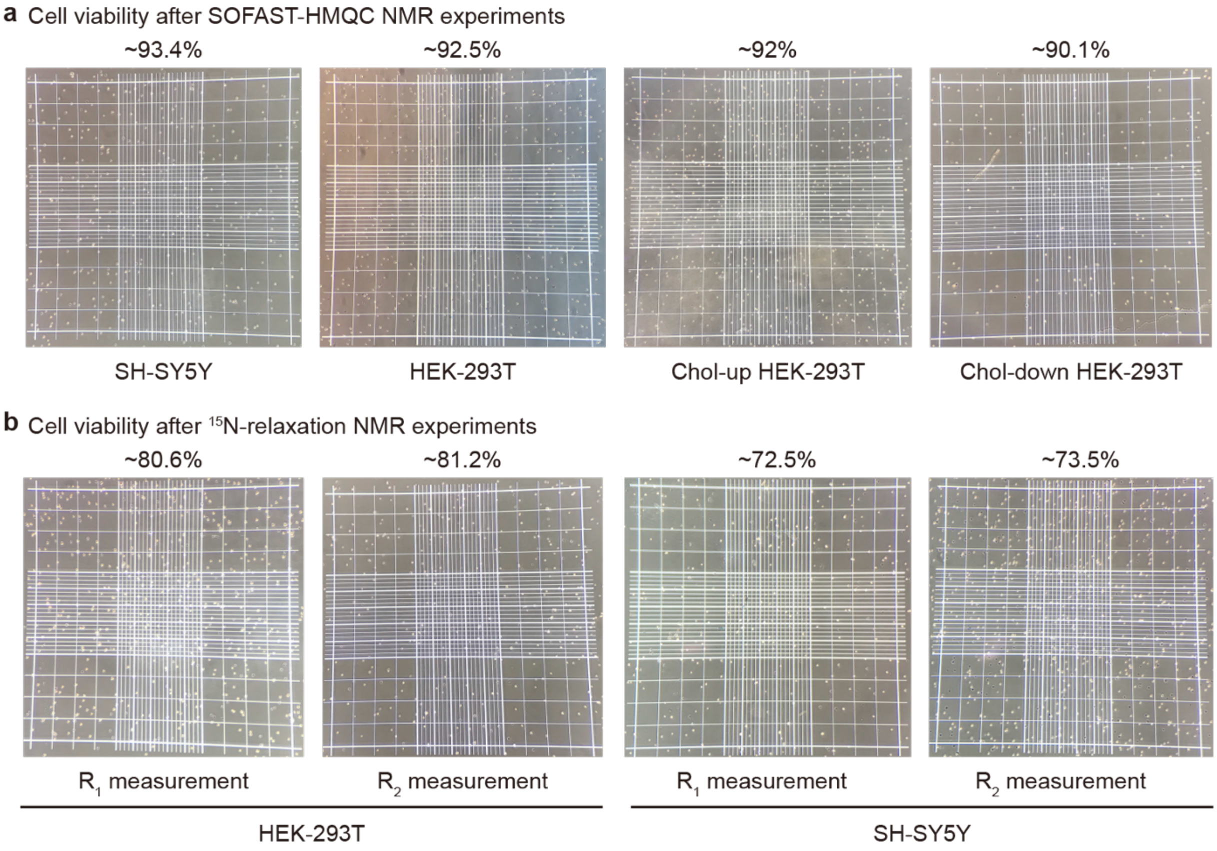

Supplementary Figure 14 | Trypan blue cell-viability tests on in-cell NMR specimens. a, Average percentages of alive cells are indicated for different cells after SOFAST-HMQC NMR experiments. Chol-up: cholesterol up-regulated cells, Chol-down: cholesterol down-regulated cells. **b**, Average percentages of alive cells are indicated for HEK-293T and SH-SY5Y cells after measuring ^{15}N-relaxation parameters of R_1 and R_2, respectively. Note that after the hours-long NMR relaxation experiments, the cell viability is relatively lower (~70%-80%), which might affect the R_1 and R_2 measurements due to protein leakage.
