# Supplementary material for: Different regions of synaptic vesicle membrane regulate VAMP2 conformation for the SNARE assembly
Source: Nat Commun. 2020 Mar 24;11:1531. doi: 10.1038/s41467-020-15270-4 (PMC7093461; doi:10.1038/s41467-020-15270-4)
Supplement: Supplementary file 3 — Reporting Summary [file 41467_2020_15270_MOESM3_ESM.pdf]

nature research

Corresponding author(s): Cong Liu

Last updated by author(s): Feb 19, 2020

Reporting Summary

Nature Research wishes to improve the reproducibility of the work that we publish. This form provides structure for consistency and transparency in reporting. For further information on Nature Research policies, see Authors & Referees and the Editorial Policy Checklist .

Statistics

For all statistical analyses, confirm that the following items are present in the figure legend, table legend, main text, or Methods section.

n/a	Confirmed	
☐	☒	The exact sample size (*n*) for each experimental group/condition, given as a discrete number and unit of measurement
☐	☒	A statement on whether measurements were taken from distinct samples or whether the same sample was measured repeatedly
☐	☒	The statistical test(s) used AND whether they are one- or two-sided<br>*Only common tests should be described solely by name; describe more complex techniques in the Methods section.*
☒	☐	A description of all covariates tested
☒	☐	A description of any assumptions or corrections, such as tests of normality and adjustment for multiple comparisons
☐	☒	A full description of the statistical parameters including central tendency (e.g. means) or other basic estimates (e.g. regression coefficient) AND variation (e.g. standard deviation) or associated estimates of uncertainty (e.g. confidence intervals)
☐	☒	For null hypothesis testing, the test statistic (e.g. *F*, *t*, *r*) with confidence intervals, effect sizes, degrees of freedom and *P* value noted<br>*Give P values as exact values whenever suitable.*
☒	☐	For Bayesian analysis, information on the choice of priors and Markov chain Monte Carlo settings
☒	☐	For hierarchical and complex designs, identification of the appropriate level for tests and full reporting of outcomes
☒	☐	Estimates of effect sizes (e.g. Cohen's *d*, Pearson's *r*), indicating how they were calculated

Our web collection on statistics for biologists contains articles on many of the points above.

Software and code

Policy information about availability of computer code

Data collection	We used Bruker standard SOFAST-HMQC pulse sequence to collect the in-cell NMR spectra.
Data analysis	We used Image J for image analysis; SPARKY and NMRpipe to analyze the NMR data.

For manuscripts utilizing custom algorithms or software that are central to the research but not yet described in published literature, software must be made available to editors/reviewers. We strongly encourage code deposition in a community repository (e.g. GitHub). See the Nature Research guidelines for submitting code & software for further information.

Data

Policy information about availability of data

All manuscripts must include a data availability statement. This statement should provide the following information, where applicable:

- Accession codes, unique identifiers, or web links for publicly available datasets
- A list of figures that have associated raw data
- A description of any restrictions on data availability

The chemical shifts of VAMP2(1-96) in HEK-293T and SH-SY5Y cells were deposited in Biological Magnetic Resonance Bank (BMRB) under accession number 50199 and 50198, respectively. The lipidomics raw data of lipid raft and non-raft samples were deposited in MetaboLights under accession number MTBLS1503.
We provided the raw data for the Fig. 1c-d,1g-h, 2c, 3b-d, 4a-c and supplementary Fig. 1, 3-5, 7-9, 13.
All relevant data are available from the authors.

Field-specific reporting

Please select the one below that is the best fit for your research. If you are not sure, read the appropriate sections before making your selection.

Life sciences study design

All studies must disclose on these points even when the disclosure is negative.

Sample size	Experiments described in this study were performed with at least 3-6 samples for each group.
Data exclusions	None
Replication	We tested > 3 times for individual experiment
Randomization	None
Blinding	None

Reporting for specific materials, systems and methods

We require information from authors about some types of materials, experimental systems and methods used in many studies. Here, indicate whether each material, system or method listed is relevant to your study. If you are not sure if a list item applies to your research, read the appropriate section before selecting a response.

Materials & experimental systems

n/a	Involved in the study
☐	☒ Antibodies
☐	☒ Eukaryotic cell lines
☒	☐ Palaeontology
☐	☒ Animals and other organisms
☒	☐ Human research participants
☒	☐ Clinical data

Methods

n/a	Involved in the study
☒	☐ ChIP-seq
☒	☐ Flow cytometry
☒	☐ MRI-based neuroimaging

Antibodies

Antibodies used	anti-α-Synuclein (BD Bioscience, Cat.# 610787); anti-GAPDH (CST, Cat.# 2118); anti-IRE1α (CST, Cat.# 14C10); anti-Mouse Alexa Fluor 594 (Invitrogen, Cat.# A-11020); anti-ACSL4 (Santa Cruz, Cat. # sc-365230); anti-Flotillin2 (Santa Cruz, Cat.# sc-28320); anti-Rabphilin3A (Santa Cruz, Cat.# 393197); anti-VAMP2 (SYSY, Cat.# 104211); anti-Oyster-550-VAMP2 (SYSY, Cat.# 104211C3); FITC-phalloidin (Yeasen, Cat.# 4073ES75)
Validation	The antibodies are well validated for the indicated use by the manufacturer available on their websites.

Eukaryotic cell lines

Policy information about cell lines

Cell line source(s)	HEK-293T (Cat.# CRL-3216) and SH-SY5Y (Cat.# CRL-2266) cell lines were purchased from ATCC, USA.
Authentication	HKE-293T and SH-SY5Y cells have been authenticated by STR method.
Mycoplasma contamination	The cell line is negative for mycoplasma contamination
Commonly misidentified lines (See ICLAC register)	No commonly misidentified cell lines were used.

Animals and other organisms

Policy information about studies involving animals; ARRIVE guidelines recommended for reporting animal research

Laboratory animals	8-week-old C57/BL6 male mice used in this paper were purchased from Lingchang Company, Shanghai
Wild animals	None
Field-collected samples	None
Ethics oversight	The mouse study was approved by the Ethic Committee of the Interdisciplinary Research Center on Biology and Chemistry, CAS, Shanghai.

Note that full information on the approval of the study protocol must also be provided in the manuscript.
