# Supplementary material for: Different regions of synaptic vesicle membrane regulate VAMP2 conformation for the SNARE assembly
Source: Nat Commun. 2020 Mar 24;11:1531. doi: 10.1038/s41467-020-15270-4 (PMC7093461; doi:10.1038/s41467-020-15270-4)
Supplement: Supplementary file 4 — Description of Additional Supplementary Files [file 41467_2020_15270_MOESM4_ESM.pdf]

Title: Supplementary Data 1.

Description: Quantitative identification of lipids in lipid-raft and non-raft regions of SV membrane. Three separately isolated lipid-raft and non-raft samples (S) were quantified by MS-based lipidomic profiling. Isomers were numbered for differentiating the lipids' name. pmol µg^-1: each quantified lipid molecular species (pmol) was normalized by total proteins of the SVs (µg). mol%: each quantified lipid molecular species (pmol) was normalized by total lipids of each sample (pmol). PC: Phosphatidylcholine; LPC: Lysophosphatidylcholine; PE: Phosphatidylethanolamine; LPE: Lysophosphatidylethanolamine; PS: Phosphatidylserine; PG: Phosphatidylglycerol; PI: Phosphatidylinositol; PA: Phosphatidic acid; Cer: Ceramide; SM: Sphingomyelines; TG: Triacylglycerols; Chol: Cholesterol.
